# Global research trends on DPP-4 inhibitors and cardiovascular outcomes: a comprehensive bibliometric analysis

**DOI:** 10.1097/MS9.0000000000003089

**Published:** 2025-03-27

**Authors:** Ehsan Amini-Salehi, Maryam Hasanpour, Abdulhadi Alotaibi, Pegah Rashidian, Seyyed Mohammad Hashemi, Amir Nasrollahizadeh, Negin Letafatkar, Parsa Saberian, Reza Amani-Beni, Najmeh Shanbehzadeh

**Affiliations:** a Guilan University of Medical Sciences, Rasht, Iran; b Department of Medicine and Surgery, Vision Colleges, Riyadh, Saudi Arabia; c Cardiovascular Research Center, Hormozgan University of Medical Sciences, Bandar Abbas, Iran; d Tehran Heart Center, Cardiovascular Diseases Research Institute, Tehran University of Medical Sciences, Tehran, Iran; e Heart Failure Research Center, Cardiovascular Research Institute, Isfahan University of Medical Sciences, Isfahan, Iran; f Department of Oral and Maxillofacial Medicine, School of Dentistry, Hormozgan University of Medical Sciences, Bandar Abbas, Iran

**Keywords:** bibliometric analysis, cardiovascular outcomes, DPP-4 inhibitors, research trends, type 2 diabetes

## Abstract

**Background:**

Dipeptidyl peptidase-4 (DPP-4) inhibitors are oral antihyperglycemic agents commonly prescribed for type 2 diabetes (T2DM). Due to the intricate relationship between glucose regulation and cardiovascular diseases (CVDs), DPP-4 inhibitors have attracted attention for their cardiovascular safety and efficacy. This bibliometric analysis aims to provide insights into the global research landscape on DPP-4 inhibitors and cardiovascular outcomes (CVOs).

**Methods:**

A bibliometric analysis was performed, using the Web of Science Core Collection. Data were analyzed using VOSviewer, CiteSpace, and Biblioshiny.

**Results:**

The United States led in publication output, followed by Japan and China. Harvard University and the University of Toronto were the leading institutions. The most influential journals were Cardiovascular Diabetology and Diabetes Obesity & Metabolism. Darren K. McGuire was the most prolific author followed by Rury R. Holman. The most commonly occurring keyword was heart failure. Cluster analysis revealed key thematic areas in the field, including “incretin-based therapy,” “dipeptidyl peptidase-4 inhibition,” and “cardiovascular safety.” Emerging clusters, such as “atrial fibrillation,” have gained attention in recent years, highlighting evolving areas of investigation.

**Conclusion:**

This study underscores the importance of CVOs in the research on DPP-4 inhibitors. The high frequency of keywords such as “heart failure,” along with recent terms like “mortality” and “risk,” highlights a strong focus on cardiovascular safety and complications in the literature. Our analysis reflected that most studies address these critical aspects of cardiovascular health, discussing the potential role of DPP-4 inhibitors in mitigating adverse outcomes, particularly in patients with T2DM.

## Introduction

For decades, diabetes has been one of the leading causes of mortality and morbidity across all countries^[1–6]^ . About 33% of diabetes melllitus (DM) patients may have cardiovascular diseases (CVDs), and the regional and global burden of CVDs attributable to DM poses significant challenges^[7–11]^. The dipeptidyl peptidase-4 (DPP-4) inhibitors represent a widely used class of oral antihyperglycemic agents primarily indicated for the management of type 2 diabetes mellitus (T2DM)^[12,13]^. By inhibiting the enzymatic degradation of incretin hormones, DPP-4 inhibitors enhance glucose-dependent insulin secretion, reduce glucagon release, and thereby promote glycemic control without causing hypoglycemia^[14–16]^. Their favorable safety profile, particularly in terms of weight neutrality and minimal risk of hypoglycemia, has contributed to their widespread use in diabetic populations^[15,17]^. CVDs remain the leading cause of death and disability in patients with T2DM^[18–20]^. The interplay between glucose metabolism and cardiovascular risk has led to a growing interest in the cardiovascular effects of glucose-lowering therapies, including DPP-4 inhibitors^[21–23]^.

**HIGHLIGHTS**

- Dipeptidyl peptidase-4 (DPP-4) inhibitors are important oral medications for managing type 2 diabetes (T2DM) and may also offer cardiovascular benefits.
- The analysis identified critical themes, such as “incretin-based therapy” and “cardiovascular safety,” with the United States, Japan, and China as the leading contributors.
- The findings highlight the importance of understanding the cardiovascular effects of DPP-4 inhibitors for improving patient outcomes in T2DM.

Given the ongoing debate about the cardiovascular safety and potential benefits of DPP-4 inhibitors, there has been a surge of scientific research focusing on this topic. A bibliometric analysis, which involves the quantitative assessment of academic literature, offers a comprehensive way to understand global research trends in this domain^[24–26]^. By mapping the volume, citation impact, authorship networks, and collaborative efforts, a bibliometric study shows key publications, influential researchers, and dominant research themes in the field of DPP-4 inhibitors and cardiovascular disorders. Furthermore, this approach enables the identification of knowledge gaps, emerging trends, and future research directions that may inform clinical practice and drug development.

This bibliometric analysis aims to evaluate the global research output on DPP-4 inhibitors in relation to cardiovascular disorders. By examining publication trends, author collaborations, and citation networks, this study aims to offer an in-depth overview of this field’s development, recognize notable individuals in its evolution, and identify promising prospects for further research.

## Methods

### Data collection

**Figure 1.**
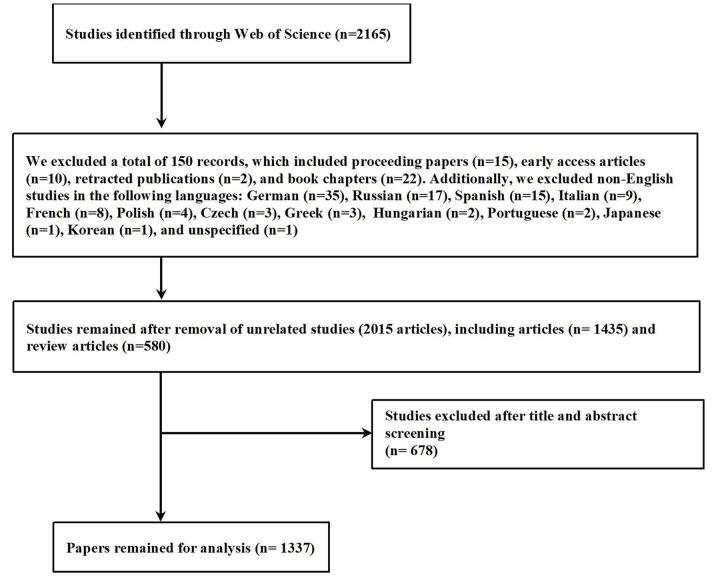
Study selection process.

On 18 August 2024, a search was carried out using the Web of Science Core Collection, which is a reliable database containing over 12 000 reputable sources^[27–29]^. To refine the search, a strategy involving several keywords was implemented (Table S1, http://links.lww.com/MS9/A750). Initially, 2165 papers were found. After excluding book chapters, editorials, conference papers, letters, and pre-publication articles, the final number of publications was narrowed down to 1337 (Fig. 1).

**Figure 2.**
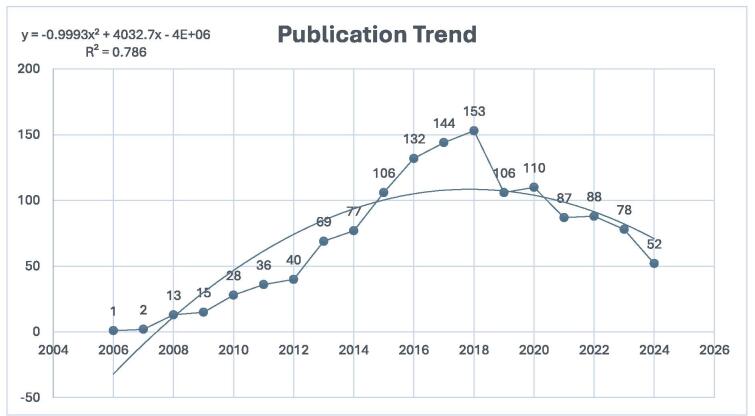
Trends in publications regarding DPP-4 inhibitors in CVOs (dotted line shows the trendline).

### Data analysis

The analysis of documents from the Web of Science Core Collection was carried out using VOSviewer, CiteSpace, and Biblioshiny (part of the Bibliometrix R package). The data were formatted into CSV and plain text files for use in these tools.

VOSviewer, a powerful tool developed by Leiden University, specializes in mapping and visualizing relationships in scholarly publications. It efficiently creates visual representations of co-citation, co-occurrence, citation, and bibliographic coupling among articles, journals, authors, institutions, countries, and keywords^[30–32]^.

CiteSpace, another essential tool, is a Java-based application that merges data visualization with scientometric analysis. By generating knowledge maps, it reveals the structural patterns, trends, and distribution of scientific knowledge, helping to highlight pivotal research, emerging topics, and important shifts in academic communication^[33]^.

Biblioshiny, the intuitive interface of the Bibliometrix R package, further facilitates bibliometric analysis by offering user-friendly access to network analysis, descriptive data insights, and visual representations of bibliometric networks, streamlining the entire research process^[34]^.

## Results

### Publication trend

The trends in publication over time offer valuable insights into the research focus within the field. Figure 2 illustrates the growing interest in DPP-4 inhibitors in cardiovascular diseases, particularly since 2006. Research activity initially started slowly, with only one publication in 2006 and two in 2007. However, from 2008, the number of publications steadily increased, reaching a peak of 153 in 2018. Since then, there has been a slight decline, with 106 papers published in 2019 and 110 in 2020. In 2021, 87 publications were recorded, followed by 88 in 2022 and 78 in 2023. As of 2024, there have been 52 publications, indicating a stabilization in the number of publications in recent years.

**Figure 3.**
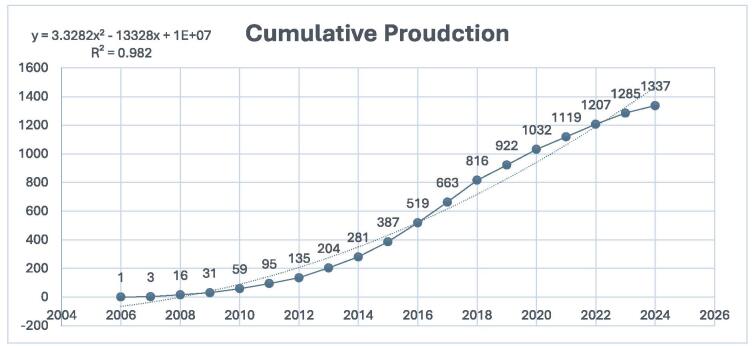
Cumulative publications on DPP-4 inhibitors in CVOs (dotted line shows the trendline).

The cumulative growth of research in DPP-4 inhibitors in CVDs has demonstrated a remarkable increase over time (Fig. 3). Initially, the early growth of research was slow, with cumulative publications remaining below 10 until 2008. However, from 2008 onwards, the field began to gain recognition, with the cumulative total reaching 387 papers by 2015. From 2016, the growth rate accelerated dramatically, with the cumulative production reaching 922 papers by 2019. This rapid expansion continued, with the total number of publications climbing to 1207 by 2022. By 2024, the cumulative total had further increased to 1337 papers, despite the year still being in progress. This steep rise highlights the growing impact and widespread interest in DPP-4 inhibitors in cardiovascular diseases, reflecting the field’s increasing significance and influence.

### Countries and institutions

**Figure 4.**
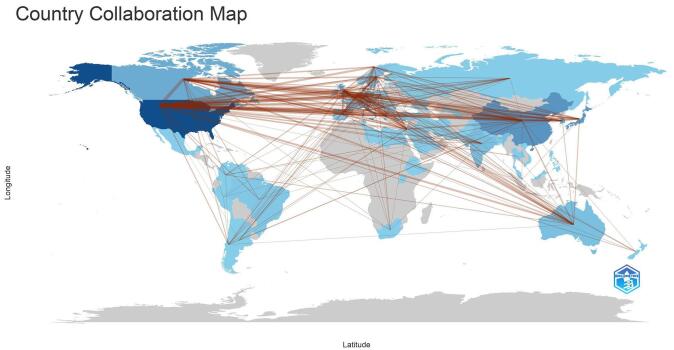
Countries’ collaboration in the field of DPP-4 inhibitors and CVOs.

The analysis of contributions by countries and institutions reveals global engagement in the field. Figure 4 shows the collaboration among countries. A total of 73 countries have made contributions, highlighting the widespread interest and collaboration. Some of the top 10 countries were the United States (449 publications), followed by Japan (180 publications) and China (135 publications). England (126 publications) and Canada (114 publications) were also key contributors. Table 1 shows the top 10 countries in terms of publications.

**Table 1:** Top 10 countries in the field of DPP-4 inhibitors in CVOs regarding publication.

Rank	Country	Number of Publications
1	USA	449
2	Japan	180
3	People’s Republic of China	135
4	England	126
5	Canada	114
6	Germany	110
7	Italy	103
8	South Korea	77
9	Taiwan	64
10	India	51

**Figure 5.**
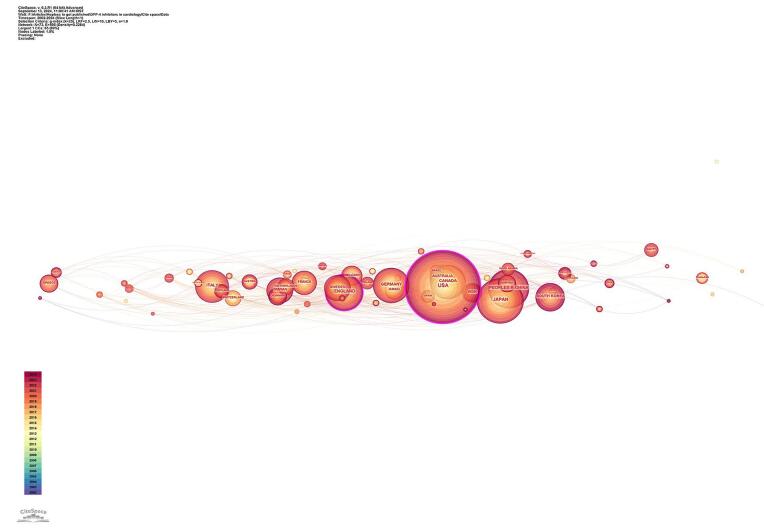
Countries with high centrality in the field of DPP-4 inhibitors and CVOs.

Centrality is a determinant showing the impact of countries in the field. The top countries based on centrality scores were the United States (0.25), followed by England (0.18) and Germany (0.09) (Fig. 5). Table 2 shows the top 10 countries regarding centrality.

**Table 2:** Top 10 countries in the field of DPP-4 inhibitors in CVOs regarding centrality.

Rank	Country	Centrality
1	United States	0.25
2	England	0.18
3	Germany	0.09
4	Canada	0.08
5	Saudi Arabia	0.08
6	South Korea	0.06
7	Sweden	0.06
8	Hungary	0.06
9	Taiwan	0.05
10	India	0.05

**Figure 6.**
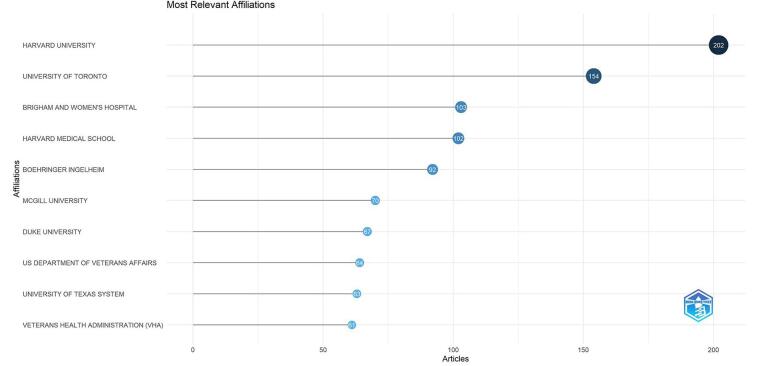
Top 10 institutions in the field of DPP-4 inhibitors in cardiovascular diseases.

A total of 1931 institutions have contributed to research in this area. Among these, the top 10 institutions are Harvard University (202 publications), followed by the University of Toronto (*n* = 154). Other leading organizations include Brigham and Women’s Hospital (*n* = 103 publications), Harvard Medical School (*n* = 102), and Boehringer Ingelheim (*n* = 92). Figure 6 shows the top 10 institutions.

### Journals and co-cited journals

**Figure 7.**
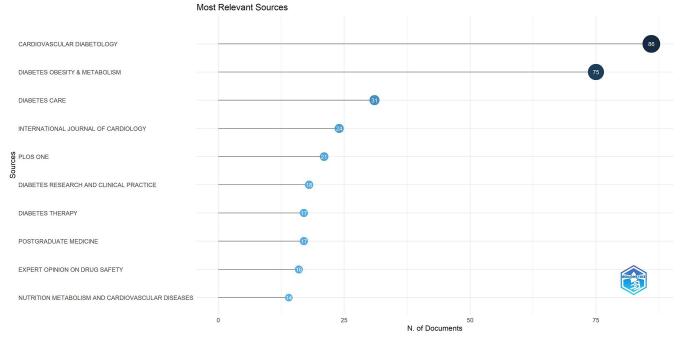
Top 10 journals in the field of DPP-4 inhibitors in CVOs.

Our analysis identified 429 sources for publications. Among these, the top 10 journals were Cardiovascular Diabetology (86 publications), followed by Diabetes Obesity & Metabolism (75 publications). Other prominent sources included Diabetes Care (31 publications), the International Journal of Cardiology (24 publications), and PLOS One (21 publications). Figure 7 illustrates the top sources and their contributions to the field.

**Figure 8.**
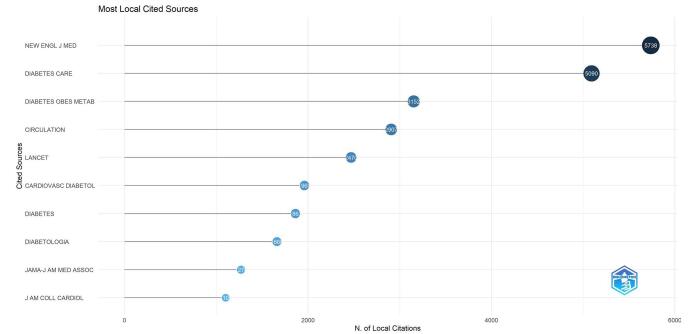
Top 10 co-cited journals in the field of DPP-4 inhibitors in cardiovascular diseases.

The analysis also identified 5071 co-cited journals. The New England Journal of Medicine had the highest number of co-citations (*n* = 5738), followed by Diabetes Care (n = 5090) and Diabetes, Obesity and Metabolism (*n* = 3152). Other frequently co-cited journals included Circulation (*n* = 2907) and The Lancet (*n* = 2470) (Fig. 8).

**Figure 9.**
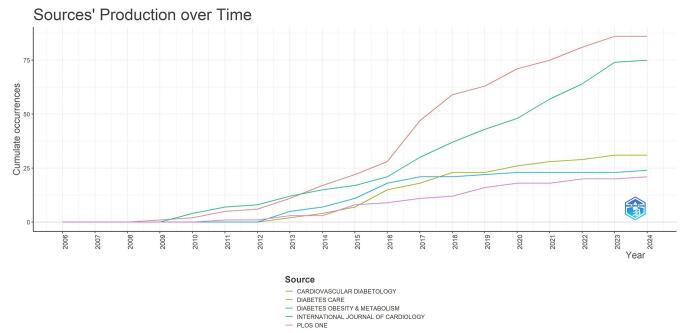
Journals’ production over time in the field of DPP-4 inhibitors in CVOs.

The analysis of publication trends over time reveals significant changes in research output across various journals. Figure 9 illustrates the cumulative number of publications from different sources, showcasing the growth and development of the field. From 2006 to 2009, research production was minimal. By 2010, Cardiovascular Diabetology, Diabetes, Obesity and Metabolism, and PLOS One emerged as active journals in the field. In the subsequent years, publication numbers increased across other journals. By 2015, Cardiovascular Diabetology, Diabetes, Obesity and Metabolism, the International Journal of Cardiology, PLOS One, and Diabetes Care were the leading journals in the field. A notable surge in publication numbers began around 2016, and by 2024, Cardiovascular Diabetology and Diabetes, Obesity and Metabolism had become the most prominent journals in the field.

### Cited and co-cited papers

**Figure 10.**
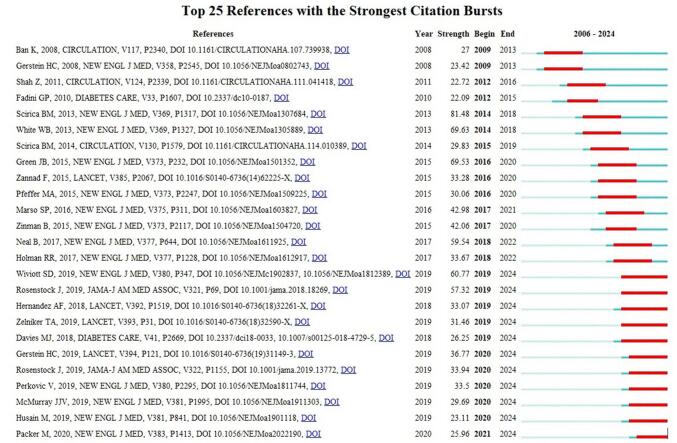
Citation burst of references in the field of DPP-4 inhibitors in CVDs.

Table 3 presents the top 10 cited and co-cited references in the field of DPP-4 inhibitors and CVOs. The most cited paper was “Saxagliptin and CVOs in Patients with Type 2 Diabetes Mellitus,” published in 2013. Following this, the second most cited paper was “Effect of Sitagliptin on CVOs in Type 2 Diabetes,” published in 2015. The third entry was “Heart failure and mortality outcomes in patients with type 2 diabetes taking alogliptin versus placebo in EXAMINE: a multicentre, randomised, double-blind trial,” published in 2015. Figure 10 shows the references with citation burst.

**Table 3:** Top 10 cited and co-cited references in the field of DPP-4 inhibitors in CVOs.

Number	Title of most cited paper	DOI	Published Year	Title of most co-cited paper	DOI	Published Year
**1**	Saxagliptin and Cardiovascular Outcomes in Patients with Type 2 Diabetes Mellitus	10.1056/nejmoa1307684	2013	Saxagliptin and Cardiovascular Outcomes in Patients with Type 2 Diabetes Mellitus	10.1056/NEJMoa1307684	2013
**2**	Effect of Sitagliptin on Cardiovascular Outcomes in Type 2 Diabetes	10.1056/nejmoa1501352	2015	Alogliptin after Acute Coronary Syndrome in Patients with Type 2 Diabetes	10.1056/NEJMoa1305889	2013
**3**	Heart failure and mortality outcomes in patients with type 2 diabetes taking alogliptin versus placebo in EXAMINE: a multicentre, randomised, double-blind trial	10.1016/s0140-6736(14)62225-x	2015	Effect of Sitagliptin on Cardiovascular Outcomes in Type 2 Diabetes	10.1056/NEJMoa1501352	2015
**4**	Heart failure, saxagliptin, and diabetes mellitus: observations from the SAVOR-TIMI 53 randomized trial	10.1161/circulationaha.114.010389	2014	Liraglutide and Cardiovascular Outcomes in Type 2 Diabetes	10.1056/NEJMoa1603827	2016
**5**	Comparative Effectiveness and Safety of Medications for Type 2 Diabetes: An Update Including New Drugs and 2-Drug Combinations	10.7326/0003-4819-154-9-201 105 030-00336	2011	Heart failure and mortality outcomes in patients with type 2 diabetes taking alogliptin versus placebo in EXAMINE: a multicentre, randomised, double-blind trial	10.1016/S0140-6736(14)62 225-X	2015
**6**	Type 2 diabetes mellitus and heart failure: a position statement from the Heart Failure Association of the European Society of Cardiology	10.1002/ejhf.1170	2018	Canagliflozin and Cardiovascular and Renal Events in Type 2 Diabetes	10.1056/nejmoa1611925	2017
**7**	Cardiovascular biology of the incretin system	10.1210/er.2011-1052	2012	Empagliflozin, Cardiovascular Outcomes, and Mortality in Type 2 Diabetes	10.1056/nejmoa1504720	2015
**8**	Effect of Linagliptin vs Glimepiride on Major Adverse Cardiovascular Outcomes in Patients With Type 2 Diabetes	10.1001/jama.2019.13772	2019	Intensive blood-glucose control with sulphonylureas or insulin compared with conventional treatment and risk of complications in patients with type 2 diabetes (UKPDS 33). UK Prospective Diabetes Study (UKPDS) Group	10.1016/s0140-6736(98)07019-6	1998
**9**	Pharmacodynamics, efficacy and safety of sodium-glucose co-transporter type 2 (SGLT2) inhibitors for the treatment of type 2 diabetes mellitus	10.1007/s40265-014-0337-y	2015	Effect of rosiglitazone on the risk of myocardial infarction and death from cardiovascular causes	10.1056/nejmoa072761	2007
**10**	Cardiovascular Actions and Clinical Outcomes With Glucagon-Like Peptide-1 Receptor Agonists and DPP-4 Inhibitors	10.1161/CIRCULATIONAHA.117.028136	2017	Heart failure, saxagliptin, and diabetes mellitus: observations from the SAVOR-TIMI 53 randomized trial	10.1161/circulationaha.114.010389	2014

In terms of co-cited papers, the most co-cited reference was “Saxagliptin and CVOs in Patients with T2DM,” published in 2013. The second most co-cited paper was “Alogliptin after Acute Coronary Syndrome in Patients with Type 2 Diabetes” published in 2013. The third one was “Effect of Sitagliptin on CVOs in Type 2 Diabetes,” published in 2015.

### Authors and co-cited authors

A total of 6,102 authors were recognized for their contributions. The analysis identified Darren K. McGuire as the leading author with the highest number of publications (34), followed by Rury R. Holman with 26 publications. Jennifer B. Green ranked third with 25 publications, while Eric D. Peterson and John B. Buse contributed 20 and 19 publications, respectively (Table 4).

**Table 4:** Top ten Cited and co-cited authors in the field of DPP-4 inhibitors in CVOs.

Number	Author with a High Number of Publications	Number of Publications	Author with a High Number of Citations	Number of Citations	The Most Co-cited Authors	Number of Citations
1	McGuire, Darren K.	34	McGuire, Darren K.	4064	Scirica, BM	823
2	Holman, Rury R.	26	Holman, Rury R.	2865	Rosenstock, J	656
3	Green, Jennifer B.	25	Buse, John B.	2844	White, WB	628
4	Peterson, Eric D.	20	Green, Jennifer B.	2697	Scheen, AJ	541
5	Buse, John B.	19	Lachin, John M.	2687	Green, JB	533
6	Bhatt, Deepak L.	18	Peterson, Eric D.	2638	Gerstein, HC	489
7	Raz, Itamar	18	Standl, Eberhard	2495	Monami, M	445
8	White, William B.	18	Armstrong, Paul W.	2372	Drucker, DJ	425
9	Scirica, Benjamin M.	16	Kaufman, Keith D.	2361	Zinman, B	413
10	Mosenzon, Ofri	16	Van De Werf, Frans	2352	Holman, RR	370

In terms of citation impact, Darren K. McGuire was the most cited author with 4064 citations. Rury R. Holman followed with 2865 citations, and John B. Buse ranked third with 2844 citations. Jennifer B. Green and John M. Lachin received 2697 and 2687 citations, respectively (Table 4).

For co-citations, Benjamin M. Scirica led the list with 823 citations, followed by Julio Rosenstock with 656 citations. William B. White received 628 co-citations, and André J. Scheen and Jennifer B. Green received 541 and 533 co-citations, respectively (Table 4).

### Keyword trends, hotspots, and cluster analysis

**Figure 11.**
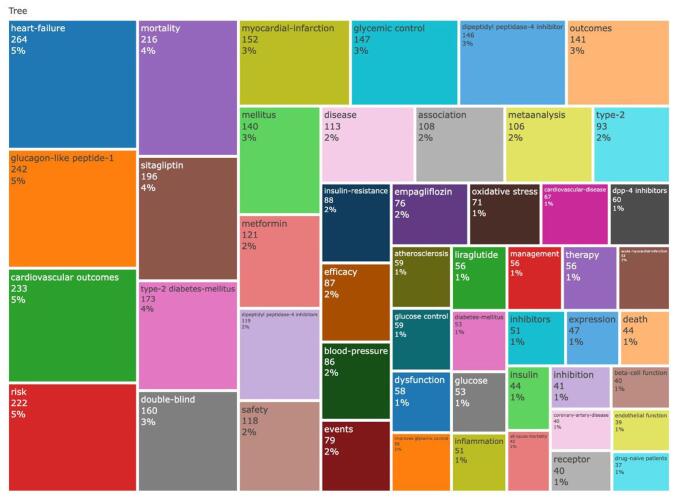
Word tree map of the keywords in the field of DPP-4 inhibitors in CVOs.

The analysis identified 3494 keywords. The most frequently occurring keywords were “heart failure” (*n* = 264), “glucagon-like peptide-1” (*n* = 242), “cardiovascular outcomes” (*n* = 233), “risk” (*n* = 222), and “mortality” (*n* = 216). Figure 11 illustrates the word tree map.

**Figure 12.**
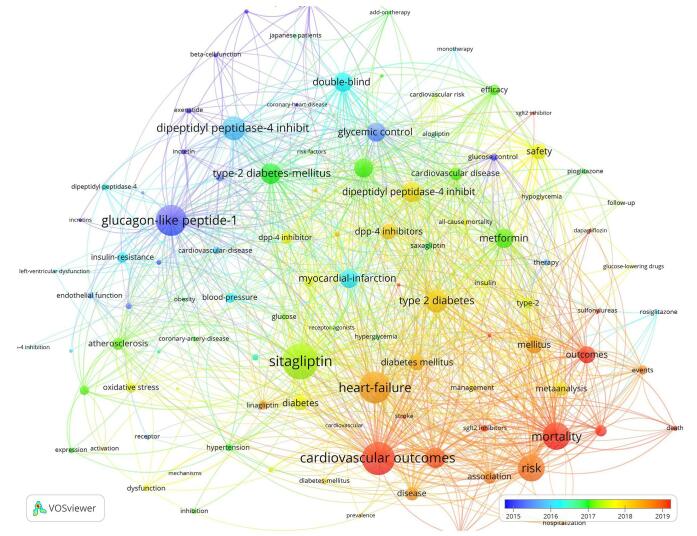
Overlay visualization of keywords in the field of DPP-4 inhibitors in CVOs.

The overlay visualization plot reveals emerging trends and recent keywords within the field. Notably, the most recent keywords include “SGLT2 inhibitors,” “Empagliflozin,” “Dapagliflozin,” “Outcomes,” “death,” and “mortality” (Fig. 12)

**Figure 13.**
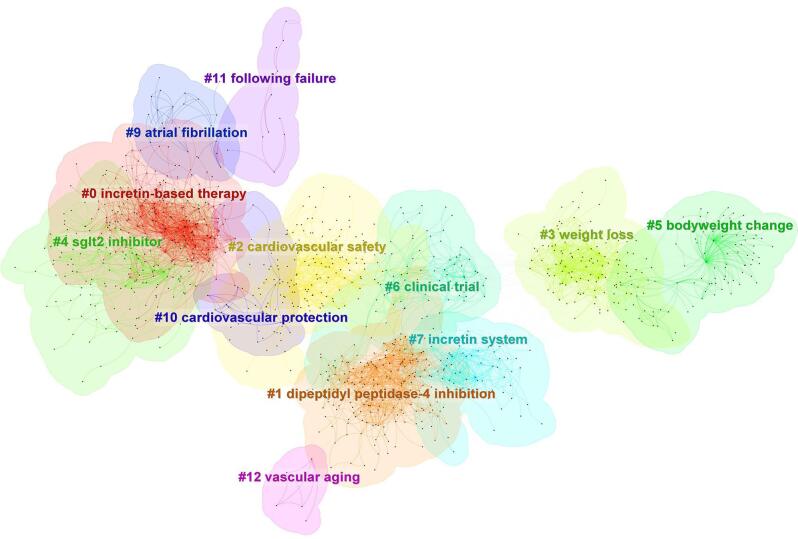
Cluster analysis of the topics in the field of DPP-4 inhibitors in CVOs.

The cluster analysis identified multiple clusters, each symbolizing a significant concept in the domain of DPP-4 inhibitors and CVOs (Fig. 13). Cluster #0 was labeled “incretin-based therapy,” while Cluster #1 concentrated on “dipeptidyl peptidase-4 inhibition.” Cluster #2 highlighted “cardiovascular safety,” and Cluster #3 focused on “weight loss.” Cluster #4 was recognized as “SGLT2 inhibitor,” with Cluster #5 emphasizing “bodyweight change.” Cluster #6 represented “clinical trial,” and Cluster #7 was associated with the “incretin system.”

**Figure 14.**
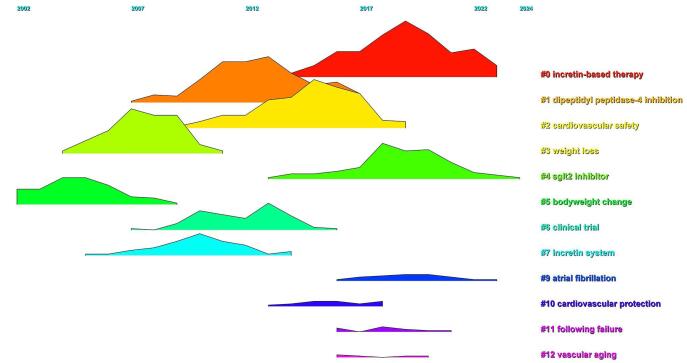
Time trend analysis of topics in the field of DPP-4 inhibitors in CVOs.

The time trend analysis of the clusters indicated that “incretin-based therapy,” “cardiovascular safety,” and “atrial fibrillation” were the most recent clusters, garnering increased attention (Fig. 14).

Regarding different DPP-4inhibitors, “Sitagliptin” was the most frequent keyword used in 336 publications. The second and third most common drugs were “Linagliptin” and “Vildagliptin,” respectively. Table 5 demonstrates the top ten DPP-4 being studied.

**Table 5:** Top 10 frequent DPP-4 inhibitors in CVOs.

Rank	Drug	Number of Publications
1	Sitagliptin	336
2	Linagliptin	81
3	Vildagliptin	76
4	Saxagliptin	71
5	Alogliptin	39
6	Teneligliptin	11
7	Gemigliptin	4
8	Evogliptin	3
9	Trelagliptin	2
10	Omarigliptin	1

## Discussion

Given the rising prevalence of CVDs in patients with T2DM, understanding the impact of DPP-4 inhibitors on CVOs has become a crucial focus of research. Numerous studies have investigated various dimensions of these drugs, including their cardiovascular effects^[3,35–45]^. This bibliometric study aims to comprehensively analyze these investigations and assess the potential role of DPP-4 inhibitors in reducing or mitigating cardiovascular risks. Given the high prevalence of CVDs among diabetic patients, understanding the potential cardiovascular effects of these inhibitors is vital.

Since 2006, there has been a marked increase in studies examining the cardiovascular effects of DPP-4 inhibitors, with a peak in publications in 2018. Although there was a slight decline afterward, the number of publications has stabilized in recent years, averaging between 80 and 110 papers per year. The field has experienced significant cumulative growth since 2016, culminating in a total of 1337 papers published by 2024. This trend underscores the growing recognition of the cardiovascular implications of DPP-4 inhibitors and highlights a sustained interest in this area, establishing it as a dynamic and significant topic in medical research.

Analysis by countries and institutions shows that the United States leads in research contributions on DPP-4 inhibitors in cardiovascular diseases, with Japan and China following in second and third place, respectively. Among institutions, Harvard University ranks first, with the University of Toronto and Brigham and Women’s Hospital in second and third positions. In terms of centrality scores, the top three countries are the United States, England, and Germany, underscoring their influential roles in driving innovation, shaping research priorities, and fostering international collaboration in advancing the study and application of DPP-4 inhibitors globally.

An assessment of the most frequently cited references reveals several pivotal studies that have significantly contributed to advancing the field. These highly cited papers provide crucial insights into various aspects of DPP-4 inhibitors in CVDs, enhancing our understanding of their effects and implications in clinical practice.

The most cited study, led by Scirica, *et al*^[46]^, titled “Saxagliptin and CVOs in Patients with T2DM,” was a randomized, double-blind, placebo-controlled trial that evaluated the cardiovascular safety of saxagliptin in 16 492 patients with T2DM at risk for cardiovascular events. The study found that saxagliptin did not significantly alter the risk of major cardiovascular events compared to placebo, meeting non-inferiority criteria but showing no cardio protective benefits. However, saxagliptin was associated with an increased risk of hospitalization for heart failure, indicating a need for alternative strategies to reduce cardiovascular risks in diabetic patients despite its effectiveness in glycemic control.

The second most cited study, conducted by Green, *et al*^[47]^, titled “Effect of Sitagliptin on CVOs in Type 2 Diabetes,” assessed the cardiovascular safety of sitagliptin in a randomized, double-blind trial involving 14 671 patients with T2DM and existing CVD. Over a median follow-up of 3 years, the study compared the addition of sitagliptin to standard care versus placebo. The findings showed that sitagliptin was non-inferior to placebo concerning the primary composite cardiovascular outcome, including cardiovascular death, nonfatal myocardial infarction, nonfatal stroke, or hospitalization for unstable angina. Furthermore, there was no significant difference in heart failure hospitalization rates between the two groups, suggesting that sitagliptin can be safely used in patients with T2DM at high cardiovascular risk without increasing the likelihood of adverse cardiovascular events.

The third most cited study by Zannad, *et al*^[48]^, titled “Heart Failure and Mortality Outcomes in Patients with Type 2 Diabetes Taking Alogliptin Versus Placebo in EXAMINE,” was a multicenter, randomized, double-blind trial evaluating the cardiovascular safety of alogliptin in patients with T2DM following a recent acute coronary syndrome. This study included 5380 participants with a median follow-up of 533 days. The results demonstrated that alogliptin was non-inferior to placebo in preventing major adverse cardiac events (MACE), such as cardiovascular death, myocardial infarction, and stroke. Additionally, there was no significant difference in heart failure hospitalization rates between the groups, indicating that alogliptin does not increase the risk of cardiovascular death or heart failure, making it a safe option for high-risk patients with T2DM and recent acute coronary events.

The overall findings of these highly cited studies indicate that research in this field has increasingly focused on the cardiovascular safety and outcomes associated with DPP-4 inhibitors in patients with T2DM. The studies specifically examine whether these medications – saxagliptin, sitagliptin, and alogliptin – affect the risk of major adverse cardiovascular events, such as heart failure, myocardial infarction, and stroke. While confirming their safety in terms of not increasing overall cardiovascular events, the research also explores specific risks, such as the increased hospitalization for heart failure associated with saxagliptin, highlighting the need for careful selection and management of these therapies in patients with varying cardiovascular risk profiles.

Keyword analysis, an essential aspect of bibliometric studies, provides insights into the main topics and emerging trends in a research field. In the study of DPP-4 inhibitors and CVOs, the most frequently used keywords are “heart failure,” “risk,” and “mortality.” “Heart failure”^[49–53]^ emphasizes the need to understand how DPP-4 inhibitors affect this serious cardiovascular condition, while “risk”^[54–58]^ highlights the potential for adverse cardiovascular events linked to these medications, underscoring the importance of evaluating their safety profiles. “Mortality”^[59–62]^ focuses on death rates as a critical endpoint, emphasizing the need to assess the overall impact of DPP-4 inhibitors on patient survival. Together, these keywords suggest a growing research focus on the adverse effects and outcomes of DPP-4 inhibitors, particularly concerning their safety and potential complications in patients with T2DM.

Overlay visualization, a valuable tool in bibliometric studies, helps researchers track the evolution of keyword trends over time and identify emerging terms within a research area. In the field of DPP-4 inhibitors and CVOs, there has been a noticeable shift toward keywords like “outcomes,” “death,” and “mortality.” This shift reflects a growing interest in understanding the broader implications of DPP-4 inhibitors on patient health, particularly their safety and long-term effects on cardiovascular health and survival rates in patients with T2DM ^[63–66]^.

Our cluster analysis in the domain of DPP-4 inhibitors and CVOs identified nine key research areas: “incretin-based therapy,” “dipeptidyl peptidase-4 inhibition,” “cardiovascular safety,” “weight loss,” “SGLT2 inhibitor,” “bodyweight change,” “clinical trial,” and “incretin system.” Among these, three clusters – “incretin-based therapy,” “cardiovascular safety,” and “atrial fibrillation” – were the most prominent, reflecting recent increases in research attention.

The “incretin-based therapy” cluster focuses on the therapeutic use of incretin hormones, such as GLP-1 and GIP, to manage blood glucose levels in T2DM while examining their potential cardiovascular benefits and risks^[16,67–69]^. The “cardiovascular safety” cluster emphasizes evaluating the safety profiles of DPP-4 inhibitors, particularly their effects on heart-related events like myocardial infarction and stroke^[70–72]^. Meanwhile, the “atrial fibrillation” cluster explores the potential links between DPP-4 inhibitors and the risk or management of atrial fibrillation – an irregular heart rhythm associated with higher risks of stroke and heart failure – highlighting it as an emerging area of interest in cardiovascular research^[35,73]^. Although the exact interplay between DM and atrial fibrillation, as well as the mechanism through which DPP4 inhibitors mitigate the atrial fibrillation risk, is not fully understood, several studies have proposed potential pathways. These include the anti-inflammatory and anti-oxidative effects of DPP-4 inhibitors, their role in modulating the electrical and mechanical properties of pulmonary veins and atria, and the ability to shorten the atrial fibrillation duration by enhancing atrial angiogenesis and activation of endothelial nitric oxide synthase^[74–78]^.

Recent studies on the CVOs of DPP-4 inhibitors have shown inconclusive results regarding their efficacy in reducing cardiovascular events in patients with T2DM. While some meta-analyses suggest a neutral effect of DPP-4 inhibitors on outcomes such as myocardial infarction, stroke, hospitalization for heart failure, and cardiovascular death, there remains uncertainty about their overall cardiovascular safety and benefits. For instance, the study conducted by Liu, *et al* (2019)^[79]^ found that the use of DPP-4 inhibitors in patients with T2DM does not significantly increase CVOs. The systematic review and meta-analysis, which included 157 478 participants, showed no significant difference in the risk of cardiovascular death, stroke, myocardial infarction, all-cause mortality, or hospitalization for cardiovascular complications between those treated with DPP-4 inhibitors and those who were not, during a follow-up period ranging from 52 to 152 weeks. These findings suggest that DPP-4 inhibitors may be safe in terms of cardiovascular events for patients with T2DM.

Also, the study by Sinha, *et al* (2019)^[37]^ found that DPP-4 inhibitors have a neutral effect on CVOs for patients with type 2 diabetes. The meta-analysis, which included multiple cardiovascular outcome trials (CVOTs), demonstrated no significant difference in the risk of myocardial infarction, stroke, combined endpoints of myocardial infarction and stroke, cardiovascular death, or hospitalization for heart failure when compared to non-users. These findings suggest that DPP-4 inhibitors neither increase nor decrease the risk of cardiovascular events, indicating their cardiovascular safety for use in these patients.

Furthermore, the study conducted by Patoulias, *et al*^[35]^, which published in 2021, found that DPP-4 inhibitors do not provide significant cardiovascular benefits for patients with T2DM. The meta-analysis of six randomized controlled trials, encompassing a total of 52 520 patients, showed that DPP-4 inhibitors had a neutral effect on the risk of major CVOs, including myocardial infarction, stroke, heart failure hospitalization, coronary revascularization, and cardiovascular death. Additionally, while DPP-4 inhibitors did not significantly affect the risk of most major cardiac arrhythmias, they were associated with a 52% increased risk of atrial flutter. Consequently, DPP-4 inhibitors are not recommended as the first-line treatment for patients with established cardiovascular disease or multiple risk factors unless newer antidiabetic drugs are not tolerated, contraindicated, or unaffordable.

The study by Chen *et al* (2022)^[36]^ is a review that discusses the potential role of DPP-4 inhibitors in cardiovascular disease management. The study highlights that while DPP-4 inhibitors have demonstrated cardiovascular safety in five recent large clinical trials, they do not show significant benefits in reducing major adverse cardiovascular events (MACE), such as cardiovascular death, non-fatal stroke, and non-fatal myocardial infarction. However, accumulating evidence suggests that DPP-4 inhibitors may offer protective effects in various cardiovascular conditions, including hypertension, calcified aortic valve disease, coronary atherosclerosis, and heart failure. The study concludes that the cardiovascular benefits of DPP-4 inhibitors require further investigation through additional prospective trials and long-term studies.

Given the inconclusive results regarding the impact of DPP-4 inhibitors on CVOs, further studies are needed to clarify their role, particularly in relation to cardiovascular risk, mortality rates, and major cardiovascular events. Also, as depicted in Fig. 2, the number of published studies on the effect of DPP-4 inhibitors on CVOs has been decreasing since 2018. It is worth mentioning that one possible cause of this decreasing trend is the underuse of different antihyperglycemic drugs in the era of SGLT-2 inhibitors and GLP-1 receptor agonists. Despite the established robust cardio-renal benefits of SGLT-2 inhibitors and GLP-1 receptor agonists across the cardiometabolic disease spectrum^[80,81]^ regarding mortality, hospitalization, and major adverse cardiovascular events, the recently published network meta-analysis by Song, *et al* demonstrated the advantages of DPP-4 inhibitors over other DM drugs in terms of improving the left ventricular end-diastolic and systolic volume^[82]^. Collectively, the role of DPP-4 inhibitors for CVOs should not be neglected and future research should focus on more extensive and long-term clinical trials to determine the precise effects of DPP-4 inhibitors on heart health and to better understand their potential benefits or risks in patients with type 2 diabetes and cardiovascular disease.

### Limitations and future suggestions

This study also had several limitations: (I) the analysis was limited to core data from the Web of Science (WoS), which may have led to the exclusion of some relevant data available in other major databases such as PubMed, Scopus, and Embase. To improve the comprehensiveness of future analyses, a multi-database approach incorporating these sources is recommended; (II) studies with incomplete or missing results were not included, which could have resulted in a potential loss of information; (III) the conclusions were drawn from a relatively small set of articles, which may impact the robustness and generalizability of the findings.

## Conclusion

This bibliometric analysis revealed significant global research efforts on DPP-4 inhibitors and their CVOs. The increasing volume of publications over the past decade reflects growing recognition of the role DPP-4 inhibitors play not only in glycemic control but also in cardiovascular risk management, particularly for patients with T2DM. Our analysis identified key research trends, with “heart failure,” “CVOs,” and “mortality” emerging as the most frequent and recent keywords, highlighting a major focus on cardiovascular safety in DPP-4 inhibitor studies.

The recent keywords like “risk” and “mortality” showed that recent studies increasingly investigated the cardiovascular benefits and risks of DPP-4 inhibitors, aligning with broader clinical concerns regarding the cardiovascular safety of glucose-lowering therapies. This suggested that DPP-4 inhibitors, due to their weight-neutral profile and minimal hypoglycemia risk, could play a beneficial role in mitigating cardiovascular events, such as heart failure and reducing mortality in T2DM patients. Moreover, the analysis revealed a continued shift toward investigating newer CVOs, such as atrial fibrillation and mortality, and comparing DPP-4 inhibitors with other glucose-lowering agents like SGLT2 inhibitors. Continued collaboration and investigation into the cardioprotective effects of DPP-4 inhibitors would be essential for informing clinical guidelines and optimizing treatment strategies for T2DM patients with high cardiovascular risk. This bibliometric approach also underscored the importance of future research focusing on evolving clusters related to CVOs, particularly with the increasing use of emerging therapeutic classes in diabetes care.

## Data Availability

The datasets used and/or analyzed during the current study are accessible from the corresponding author on reasonable request.

## Research registration unique identifying number (UIN)

The study is a bibliometric study and UIN is not applicable.
